# Influence of warm acupuncture on gut microbiota and metabolites in rats with insomnia induced by PCPA

**DOI:** 10.1371/journal.pone.0267843

**Published:** 2022-04-28

**Authors:** Hong Yu, Hui Yu, Lengge Si, Husileng Meng, Wensheng Chen, Zhanli Wang, A. Gula

**Affiliations:** 1 College of Traditional Chinese Medicine, Beijing University of Chinese Medicine, Beijing, China; 2 School of Nursing, Baotou Medical College, Baotou, China; 3 Inner Mongolia Key Laboratory of Disease-Related Biomarkers, Baotou Medical College, Baotou, China; 4 Mongolian Medicine School, Inner Mongolia Medical University, Hohhot, Inner Mongolia, China; Monash University Malaysia, MALAYSIA

## Abstract

**Background:**

Insomnia is the most common of the sleep disorders. Current pharmacotherapy treatment options are usually associated with adverse effects and withdrawal phenomena. Therapeutic alternatives with a more favorable safety profile for patients are needed. Mongolian medical warm acupuncture (MMWA) is an emerging therapeutic option for treating insomnia. However, the underlying mechanisms responsible for the anti-insomnia efficacy of the MMWA remain unclear. This study aims to investigate the effect of the MMWA on the alterations of the gut microbiota and serum metabolome in rats with insomnia.

**Results:**

We found that the relative abundances of gut bacteria and the concentrations of several serum metabolites were obviously altered in PCPA-induced insomnia rats. The MMWA treatment exerted an anti-insomnia effect. In addition, the dysbiosis of the gut microbiota and the serum metabolites were ameliorated by the MMWA. Correlation analysis between the gut microbiota and metabolites suggested that the levels of Amide c18, Benzoyl chloride, Cytosine, and N, n-dimethylarginine were positively correlated with the relative abundance of *Clostridium XlVa* and *Blautia*, which characterized the insomnia rats. KEGG enrichment analysis identified the cAMP signaling pathway involving anti-insomnia effect of the MMWA. Moreover, the MMWA intervention significantly increased contents of butyrate in feces, while effectively inhibited the expression level of GAT-1 in brain tissues.

**Conclusion:**

This study reveals that the MMWA intervention might have a major impact on the modulation of host gut microbiota and metabolites, which in turn have a crucial role in the regulation of the host’s signaling pathways associated with insomnia. The present study could provide useful ideas for the study of the intervention mechanisms of the MMWA in insomnia rat models.

## Introduction

Insomnia is the most prevalent sleep disorder, which affects 6–10% of the general population worldwide [1]. It is characterized by difficulty with sleep initiation, maintenance, or early morning awakening, and accompanied by daytime impairment. Accumulating evidence indicated that insomnia was associated with a wide range of negative health consequences, such as mental disorder, cardiovascular dysfunction, type 2 diabetes, and obesity [2–4]. Many studies have suggested that multifactorial causes are involved in the etiology of insomnia, and the detailed pathological aspects of insomnia remain unclear [5].

The gut microbiota and its metabolites have long been recognized as a key component involved in various diseases [6]. Dysbiosis of the gut microbiota perturbs the host metabolism and immune balance, leading to the occurrence of diseases [7]. Several studies have also provided preliminary support for an association between gut microbiota and sleep disorder. It was reported that the structure, composition, and function of the gut microbiota, as well as the bacterial interaction network were significantly changed in insomnia patients [8]. In another study with acute and chronic insomnia patients between 26–55 years, the microbial richness and diversity were lower with the depletion of short-chain fatty acid (SCFA)-producing bacteria [9]. These studies provide a theoretical basis for the application and development of novel strategies for insomnia therapy.

Mongolian medicine, one of the traditional medicines, showed a favorable safety profile and beneficial therapeutic effects on insomnia [10]. In Mongolian medicine, warm acupuncture is one of the main treatment methods for insomnia. It prevents and treats diseases by stimulating specific anatomic sites (acupoints) using warm silver needles [11]. It has been widely accepted by insomnia patients due to its advantages, such as efficiency, safety, and no drug dependence [12]. In a recent study, acupuncture inhibited the neuroinflammation in a mouse model of Parkinson’s disease by modulating gut microbial dysbiosis [13]. Another study reported that acupuncture might be beneficial for insomnia through modulating the gut microbiota to regulate the host inflammatory response [14]. However, the role of the gut microbiota and its metabolites on the effects of the Mongolian medicine warm acupuncture (MMWA) in insomnia is still unknown.

Our study aims to investigate the effects of the MMWA on the gut microbiota and metabolites in p-chlorophenylalanine (PCPA)-induced insomnia rats, which may help to elucidate the biological mechanisms of the gut microbiota-mediated sleep regulation.

## Methods

### Ethics

Ethical approval was obtained from Baotou Medical College Research and Ethical Review Committee (no. 2021039). At the end of the experiment, the animals were euthanized using 2% pentobarbital sodium (prepared in normal saline, 40 mg/kg, intraperitoneal injection.

### Animals and PCPA-pretreated rat model

Six-week-old male Sprague-Dawley (SD) rats (body weight, 160 to 180 g) were obtained from Vital River Laboratory Animal Technology Co., Ltd. (Beijing, China). Animals were housed individually in a temperature-controlled room (24 ± 2°C) under a 12/12-h light-dark cycle. The rats were allowed free access to food and water throughout the experiment. After one week of acclimatization, animals were randomly allocated into three groups: the control group (n = 8), the model group (n = 8), and the treatment group (n = 8). For the PCPA-induced insomnia model, rats received PCPA (300 mg/kg, dissolved in saline) via intraperitoneal injection in the morning of each day for 3 consecutive days [15]. The vehicle control group received an injection of the same volume of normal saline.

### Acupuncture treatment

In the treatment group, rats were treated with the MMWA on the acupoints of Dinghui, Heyi, and Xin acupoints once daily for seven consecutive days. Detailed acupoint locations were shown in S1 Fig. Acupoint Dinghui has been used to alleviate daftness, dizziness, and headache. Acupoint Heyi is often used to treat palpitation, agitation, and insomnia. Acupoint Xin is stimulated to alleviate delirium, anorexia, and insomnia in traditional Mongolian medicine [16]. Recent studies have verified that stimulating these three acupoints improves the sleep quality of rats with PCPA-induced insomnia [11, 12]. The acupoints were stimulated with the MMWA for 15 min each time at ~40°C with the instrument of Mongolian Model MY-I electric heating needle warmers (Shanghai, China). The needle was evenly inserted 5 mm depth for each acupoint as described previously [15]. In the other groups, these three acupoints without acupuncture treatment were slightly lifted and twisted under the same conditions. Rats were weighed before the establishment of the insomnia model and at the end of the experiment.

### Open field test

The rats were acclimated to the environment for 10 min before being subjected to an open-field test. Then, rats were placed in the center of a wooden open field box. The video tracking analysis system was used to record rat activity within the 5 min for calculating exercise distance, exercise time, and number of arm lifting.

### Sample collection

At the end of the experiment, the animals were euthanized using 2% pentobarbital sodium (prepared in normal saline, 40 mg/kg, intraperitoneal injection). Fresh feces of rats were collected directly into the sterilization centrifuge tube and were immediately stored in liquid nitrogen, then transferred to −80°C refrigerator for determination of intestinal flora. Blood was collected from the abdominal aorta. Serum was collected in a refrigerator at −80°C for serum metabolomics analysis or neurotransmitters determination. The whole brain was quickly removed on ice, and then the brain stem was separated by precision forceps. The isolated brain stem segment was quickly frozen in liquid nitrogen and stored at -80°C for later use.

### Enzyme-linked immunosorbent assay (ELISA) analysis

The neurotransmitter array kits (Absci, MD, USA) were used to detect serotonin (5-HT), norepinephrine (NE), acetylcholine (ACh), and gamma-aminobutyric acid (GABA) in serum samples according to the manufacturer’s instructions. Moreover, the concentration of cAMP was determined using an ELISA kit according to the manufacturer’s protocol [17]. The 5-HT, NE, Ach, GABA, and cAMP contents were measured at 450 nm on a microplate reader.

### Gut microbiota analysis

The total microbial DNA was extracted from fecal samples using the MagPure Stool DNA KF kit B (Magen, China). The extracted DNA was quantified with a Qubit Fluorometer by using Qubit dsDNA BR Assay kit (Invitrogen, USA), and DNA quality was examined by 1% agarose gel electrophoresis (Solarbio, Beijing, China). The V3–V4 hypervariable regions of the bacterial 16S rRNA gene were amplified by PCR with the primers 341F (5’-ACT CCT ACG GGA GGC AGC AG-3’) and 806R (5’- GGA CTA CHV GGG TWT CTA AT-3’). PCR reactions were performed using the following program: 3 min of denaturation at 94°C, 30 cycles of 30 sec at 94°C, 45 sec for annealing at 56°C, 45 sec for elongation at 72°C, and final extension at 72°C for 10 min. PCR products were purified using the AmpureXP beads (Beckman Coulter, Fullerton, CA, USA) according to the manufacturer’s instructions and quantified by the Agilent 2100 bioanalyzer (Agilent, USA). Then, the validated libraries were used for sequencing on the Illumina MiSeq platform (BGI, Shenzhen, China) according to the standard protocols.

Raw fastq files were quality-filtered to remove adaptors and low-quality and ambiguous bases and then merged by the Fast Length Adjustment of SHort reads program (FLASH, version 1.2.11) [18]. Operational units (OTUs) were clustered with a cutoff value of 97% using UPARSE (version 7.0.1090) [19]. The taxonomy of each 16S rRNA gene sequence was classified using Ribosomal Database Project (RDP) Classifier algorithm (version 2.2) using a confidence threshold of 60%. Alpha and beta diversity were calculated by MOTHUR (version 1.31.2) and QIIME (version 1.8.0) at the OTU level, respectively. The linear discriminant analysis (LDA) or LEfSe cluster was performed by LEfSe software. Phylogenetic Investigation of Communities by Reconstruction of Unobserved States (PICRUSt) based on Kyoto Encyclopedia of Genes and Genomes (KEGG) database was used to predict the microbiota functions [20].

### Metabolomics analysis

After being fully thawed at room temperature, 100 μL serum samples were extracted three times by directly adding 300 μL of precooled methanol and acetonitrile (2:1, v/v). The mixture was vortexed for 1 min and incubated at -20°C for 2 h. The samples were then centrifuged at 4000 rpm for 20 min at 4°C and the supernatant was transferred for dryness by vacuum centrifugation. The metabolites were redissolved in 150 μL of 50% cold methanol and centrifuged at 4000 rpm for 30 min. The supernatant was transferred into autosampler vials for metabolism analysis. A quality control (QC) sample was prepared by pooling the same volume of each sample to monitor the stability of the instrument.

This experiment used the ultra-performance liquid chromatography (Waters 2D UPLC, Waters, USA) tandem high-resolution mass spectrometer (Q Exactive, Thermo Fisher Scientific, USA) for separation and detection of metabolites. The separation was performed on a Waters ACQUITY UPLC BEH C18 column (100 mm × 2.1 mm, 1.7 μm, Waters, USA), which was maintained at 45°C. The mobile phase was composed of 0.1% formic acid (A) and acetonitrile (B) in the positive mode and was composed of 10 mM ammonium formate (A) and acetonitrile (B) in the negative mode. The gradient elution conditions were as follows: 0–1 min, 2% B; 1–9 min, 2%-98% B; 9–12 min, 98% B; 12–12.1 min, 98% B to 2% B; and 12.1–15 min, 2% B. The flow rate was 0.35 mL/min and the injection volume was 5 μL. The mass spectra conditions were as follows: spray voltage, 3.8/−3.2 kV; aux gas heater temperature, 350°C; capillary temperature, 320°C; sheath gas flow rate, 40 arbitrary units; aux gas flow rate, 10 arbitrary units. The full scan range was 70–1050 m/z with a resolution of 70000.

The original chromatographic peak data were processed using the Compound Discoverer 3.1 (Thermo Fisher Scientific, USA) software, which mainly included over-lapping peak resolution, peak alignment, and compound identification. Data pre-processing, statistical analysis, metabolite classification annotations, and functional annotations were performed using the metabolomics R package metaX [21] and the metabolome bioinformatic analysis pipeline [22].

### Correlation analysis between metabolites and gut microbiota

Spearman correlation analysis was performed to reveal the correlation between two variables (such as metabolite level and microbial abundance). Sparse generalized canonical correlation analysis was used to calculate the overall correlation between metabolites and microorganisms.

### Butyrate determination

Approximately 100 mg of fresh fecal contents was weighed and then mashed in acidic water (pH = 2.4). The sample was centrifuged at 12,000 g for 20 min at 4°C. The supernatants were then taken for analysis as previously described [23].

### Administration of butyrate

The insomnia model was induced by PCPA as described previously. Sodium butyrate (Sigma Aldrich, Carlsbad, CA, USA) was dissolved in physiological saline and was intraperitoneally administrated to rats at a dose of 0.6 g/kg for seven consecutive days. Animal groups included the following: PCPA-treated rats with vehicle administration (Vehicle) (n = 8) and PCPA-treated rats with sodium butyrate administration (Butyrate) (n = 8). At the end of the experiment, the animals were euthanized using 2% pentobarbital sodium and the concentration of cAMP in the brain was determined using the ELISA method [17].

### Western blot analysis

Western blot was used to examine the expression of gamma-aminobutyric acid transporter-1 (GAT-1) in the brain tissues. Frozen tissues were homogenized and total protein was extracted. Protein concentrations were measured using the BCA method. The supernatant samples containing 20 μg of protein were electrophoresed in 10% SDS polyacrylamide gels, transferred to polyvinylidene difluoride membrane (Millipore, Bedford, MA), blocked with 5% milk, and incubated overnight at 4°C with anti-GAT-1 primary antibody (1:1000, Cell Signaling Technology, MA, USA). Then the membranes were washed and incubated with the HRP-conjugated secondary antibody (1:5000; Santa Cruz Biotechnology, CA, USA), and visualized by enhanced chemiluminescence.

### Statistical analysis

SPSS 21.0 software was used to analyze the data. The significant differences between multiple groups were analyzed using one-way ANOVA, and two groups were calculated using Student’s unpaired t-test. Results are expressed as means ± SD, and a value of P < 0.05 is considered as statistically significant.

## Results

### MMWA treatment ameliorates insomnia induced by PCPA in rats

**Fig 1:**
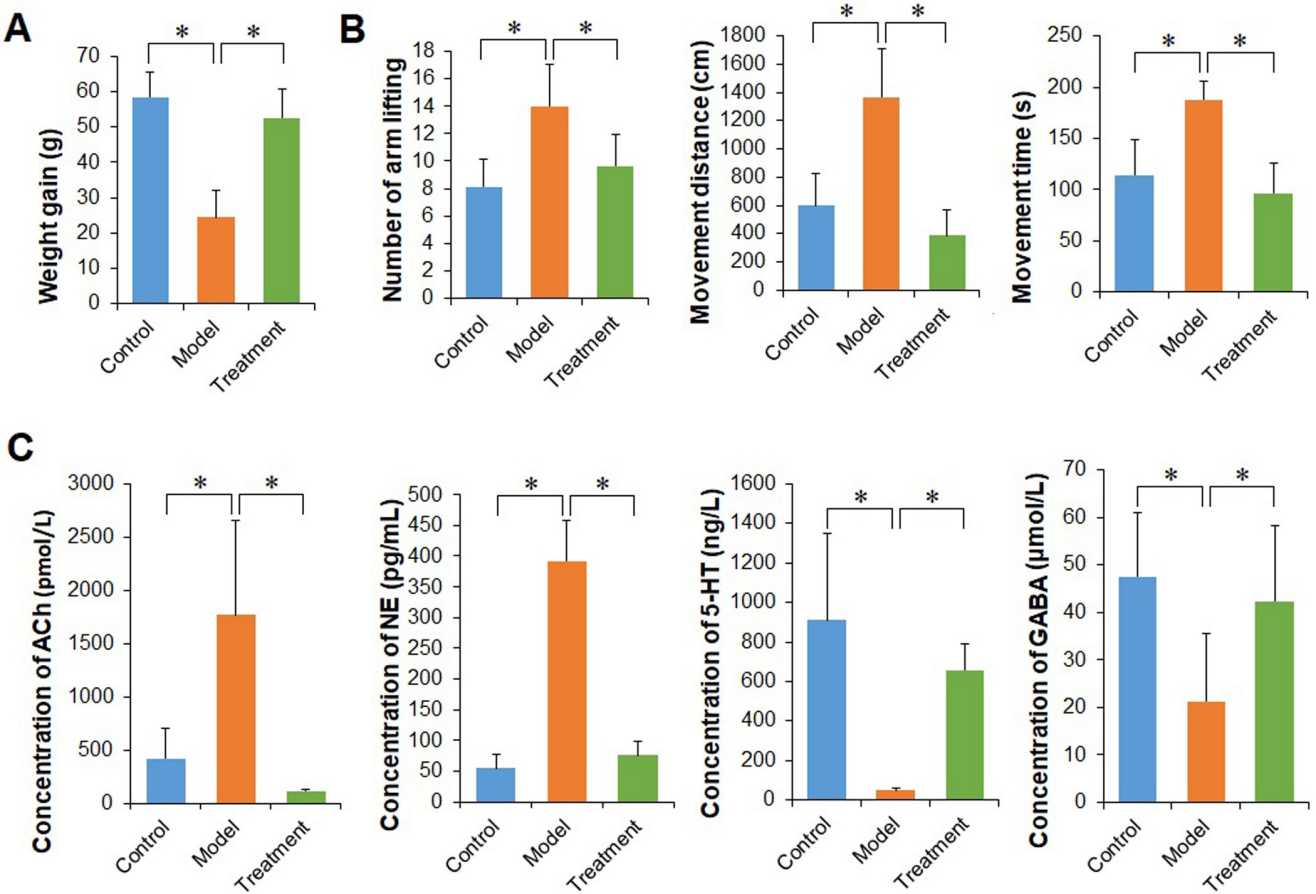
The therapeutic effect of the MMWA on insomnia rats. (A) Comparison of body weight gain of rats among three groups. (B) Comparison of the movement distance, movement time, and the number of arm lifting of rats among three groups. (C) Comparison of neurotransmitters in the serum of rats among three groups. Values are expressed as the means ± SD, n = 8 rats in each group (*P < 0.05).

As shown in Fig 1A, the insomnia models exhibited less food intake and lower weight gain when compared with the control group (P < 0.05). Treatment with the MMWA was able to substantially increase weight gain (P < 0.05) (Fig 1A). The open field test demonstrated that the rats in the model group showed an increase in the movement distance, movement time, and the number of arm lifting compared with the controls (P < 0.05). These autonomous activity test indexes trended to decrease in the treatment group (P < 0.05) (Fig 1B). As shown in Fig 1C, the rats in the model group showed lower levels of 5-HT and GABA, and higher contents of ACh and NE compared with the controls (P < 0.05). Conversely, the treatment group showed higher levels of 5-HT and GABA, and lower contents of ACh and NE than the model group (P < 0.05). These results indicated that the MMWA had a therapeutic effect on insomnia.

### MMWA treatment improves gut microbiota in PCPA-induced insomnia rats

**Fig 2:**
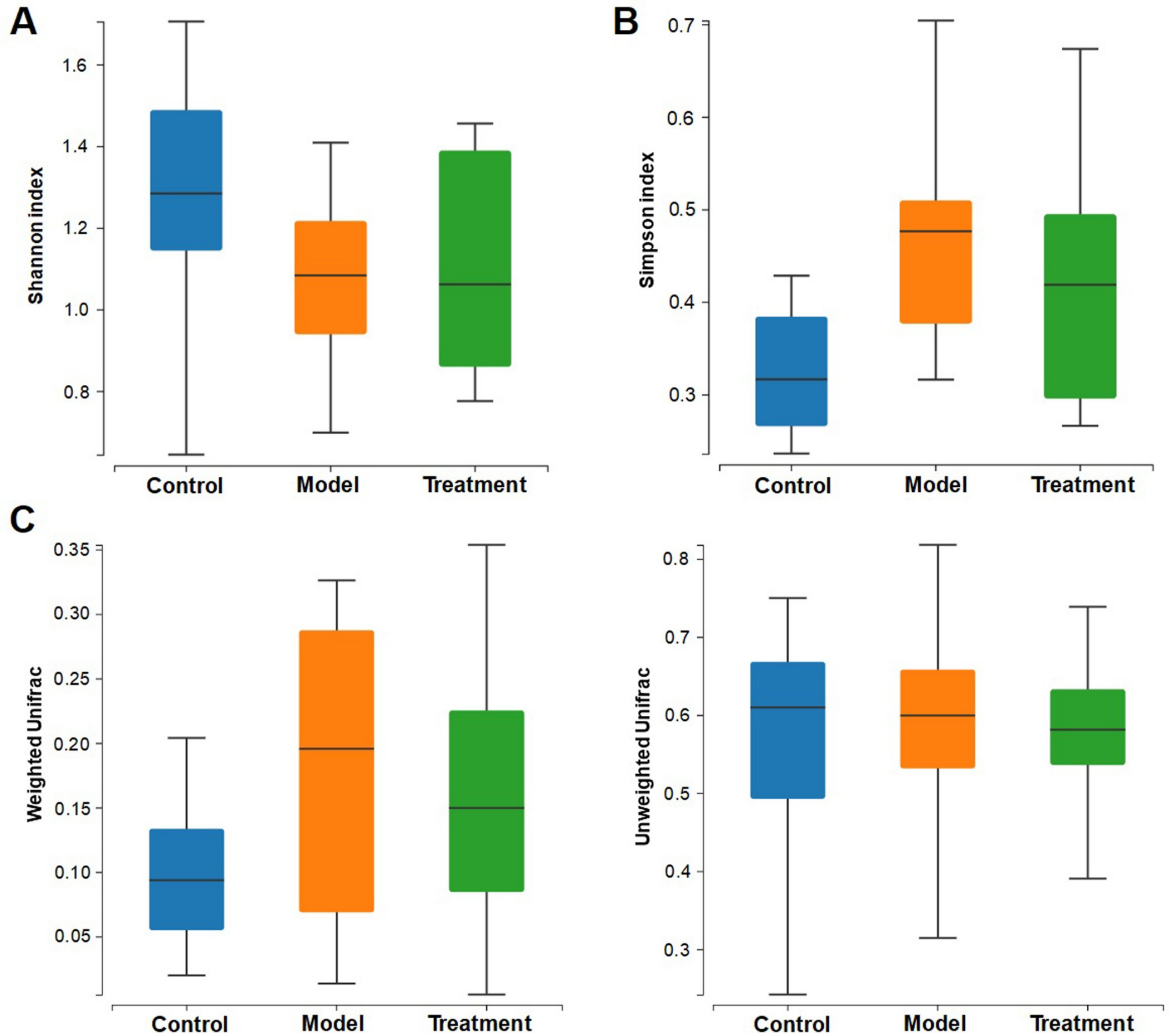
Influence of the MMWA treatment on gut microbiota diversity. (A) Shannon index. (B) Simpson index. (C) Beta diversity of gut microbiota.

The fecal microbial composition was determined by bacterial 16S rRNA sequencing. A total of 1,632,128 reads of 16S rRNA gene were retained and each sample totaled between 68,192 and 70198 (S1 Table). In addition, a total of 1513 OTUs were identified from all samples (S2 Table). Species accumulation curve based on the OTUs reached saturation, indicating that the sequencing data were sufficient to cover the majority of microbe species (S2A Fig). The rank abundance curve representing species abundance and distribution evenness was shown in S2B Fig. Fig 2 showed the diversity of gut microbiota by the alpha diversity indices. The Shannon index reflecting the species richness and evenness was 1.29 ± 0.30, 1.07 ± 0.21, and 1.10 ± 0.28 in the control, model, and treatment groups, respectively (Fig 2A). Similarly, the Simpson index reflecting community evenness in the three groups was 0.35 ± 0.12, 0.47 ± 0.11, and 0.42 ± 0.13, respectively (Fig 2B). A significant difference was observed in beta diversity based on the weighted_unifrac (P = 0.008) but not unweighted_unifrac (P = 0.817) among the three groups (Fig 2C).

**Fig 3:**
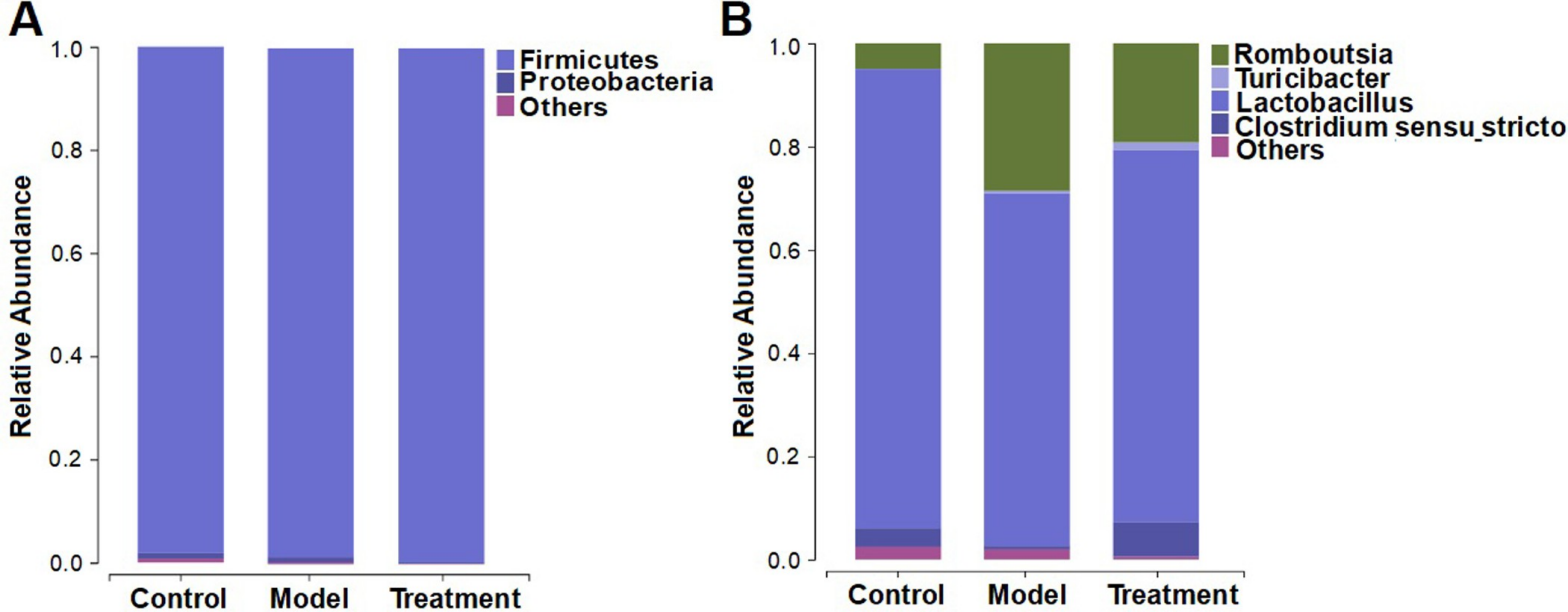
Influence of the MMWA treatment on gut microbiota composition. (A) Taxonomic composition at the phylum level. (B) Taxonomic composition at the genus level.

The microbial compositions at the phylum level were shown in Fig 3A. *Firmicutes* were the most abundant bacterial group, accounting for an average of 98.1%, 98.7%, and 99.7% sequences in the control, model, and treatment groups, respectively. Meanwhile, *Proteobacteria* represented the second dominant bacterial community in the three groups, accounting for an average of 1.1%, 1.0%, and 0.2% sequences, respectively. Other phyla were detected at low relative abundances (less than 3%). At the genus level, the microbial compositions were shown in Fig 3B. *Romboutsia*, *Lactobacillus*, *Clostridium sensu stricto* and *Turicibacter* were abundant in the three groups. Compared with the control group, a significant increase in the *Romboutsia* counts and reduction in *Lactobacillus* and *Clostridium sensu stricto* were observed in the fecal samples from the model group (P < 0.05). In contrast, the abundances of these genera were reversed by the MMWA intervention.

**Fig 4:**
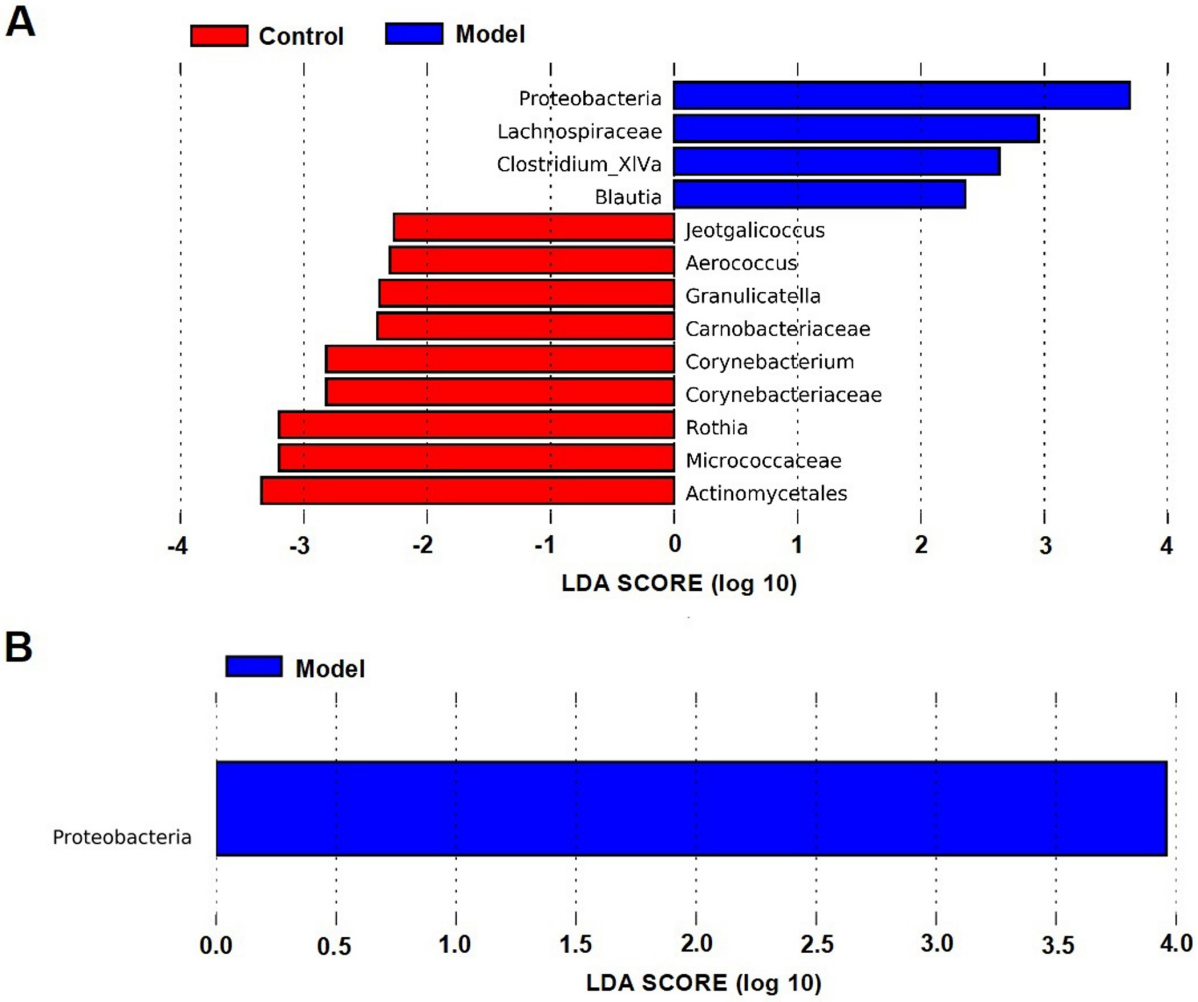
The significantly enriched bacterial taxa in different groups as determined by LEfSe analysis. (A) Pairwise taxonomic LEfSe analysis of the control and the model groups. (B) Pairwise taxonomic LEfSe analysis of the model and the treatment groups. (LDA score > 2.0, P < 0.05).

According to the LDA effect size (LEfSe) analysis, 13 taxa were significant different between the model group and the control group: 9 for the control group and 4 for the model group (Fig 4A). The control rats primarily showed higher enrichment of *Aerococcus*, *Corynebacteriaceae*, *Carnobacteriaceae*, *Corynebacterium*, *Micrococcaceae*, *Rothia*, *Jeotgalicoccus*, *Granulicatella*, and *Granulicatella*, whereas the model rats were mainly characterized by higher abundance of *Proteobacteria*, *Clostridium XlVa*, *Blautia*, and *Lachnospiraceae*. Similarly, the differences between the model group and the treatment group were also identified (Fig 4B). We observed that the *Proteobacteria* was significantly affected by the MMWA treatment. However, the proportion of *Blautia* in the MMWA-treated rats was closer to that in the model group (S3 Fig), indicating that the warm acupuncture intervention did not regulate the structure of intestinal flora to reach a healthy state.

### MMWA treatment regulates serum metabolites in PCPA-induced insomnia rats

**Fig 5:**
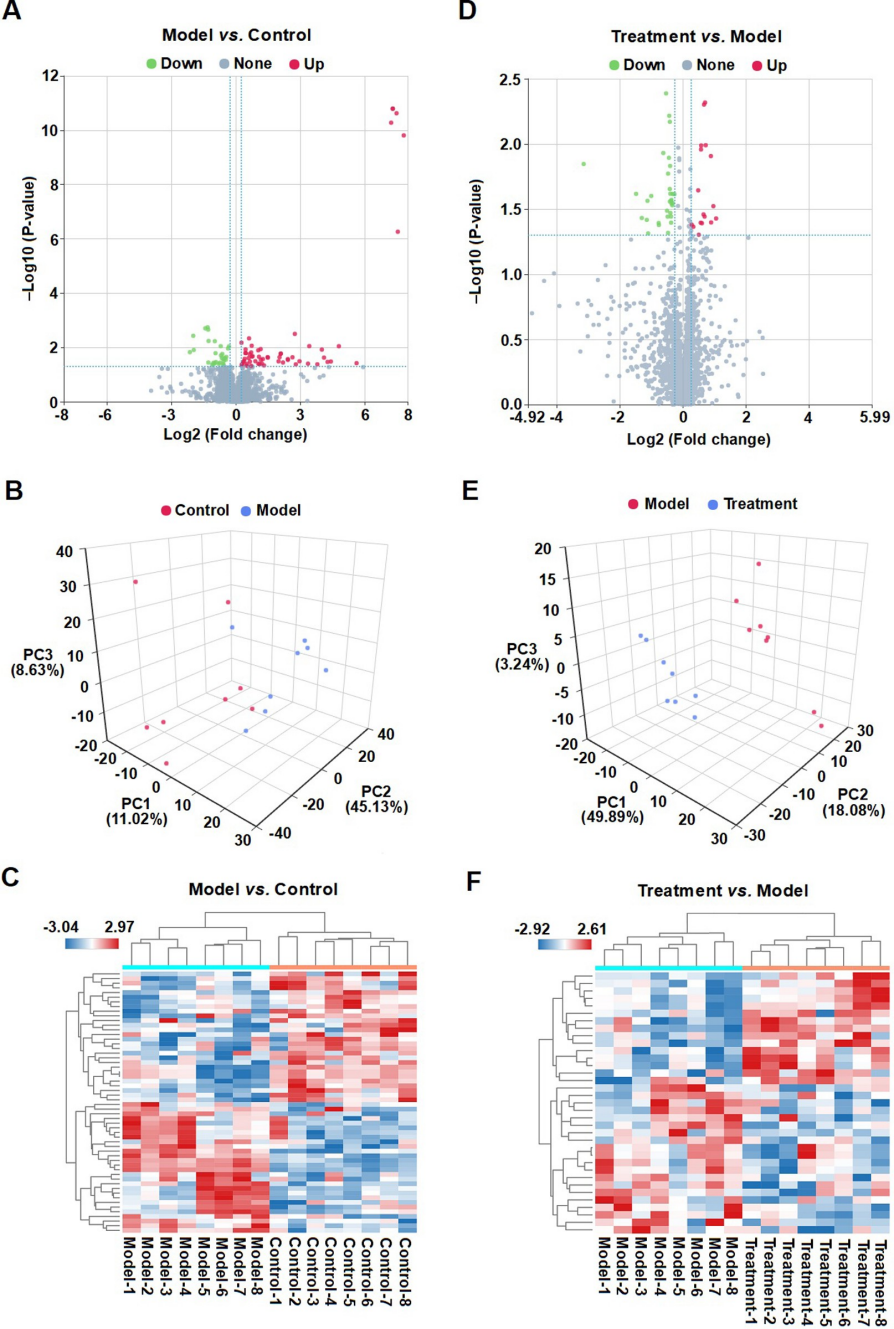
Influence of the MMWA treatment on the levels of serum metabolites. (A) Volcano plot of differential metabolites between the control group and the model group. (B) OPLS-DA score plots of serum samples from the control group and the model group in positive ion mode. (C) Heatmap analysis of differential metabolites between the control group and the model group. (D) Volcano plot of differential metabolites between the model group and the treatment group. (E) OPLS-DA score plots of serum samples from the model group and the treatment group in positive ion mode. (F) Heatmap analysis of differential metabolites between the model group and the treatment group.

The metabolite profiles in both positive and negative ion modes were identified. Because a high baseline in the negative ion mode was observed (S4 Fig), the positive ion mode was selected for further analysis in this study. A total of 88 metabolites were annotated between the control group and the model group (Fig 5A). Score plots of OPLS-DA and Heatmap showed that the model rats could be separated from the control rats (Fig 5B and 5C). Among the 88 metabolites, the levels of 57 metabolites significantly increased in the model group compared to the control group, whereas the other 31 metabolite levels dramatically decreased (S3 Table). In contrast, a total of 35 metabolites were annotated between the treatment group and the model group (Fig 5D). The treatment group could also be separated from the model group (Fig 5E and 5F). The results showed that the contents of 6 metabolites were reversed after treatment of the MMWA, including Biochanin a, Asarone, Succinic anhydride, Carbaryl, Oleoylethanolamide, and Sulfosalicylic acid (S4 Table).

**Fig 6:**
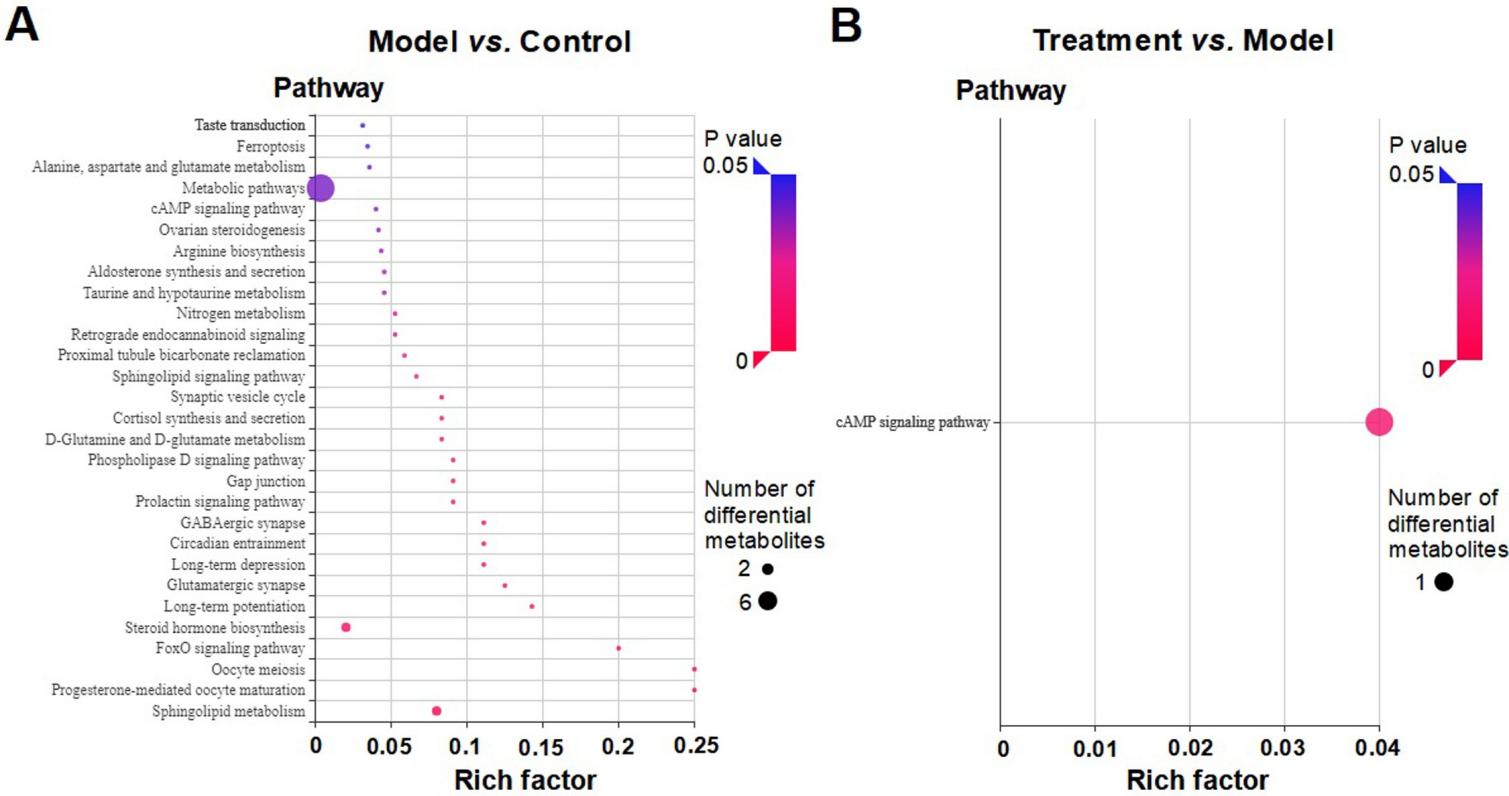
KEGG enrichment analysis of differential metabolites between the control group and the model group (A), and between the model group and the treatment group (B). The X-axis indicates rich factor and the Y-axis represents the terms of the KEGG pathways. The size of circle indicates the number of differential metabolites and the color of circle represents P-value.

KEGG pathway enrichment analysis showed that the occurrence of insomnia was associated with multiple pathways, among which the metabolic pathways and signaling pathways were considered to be especially important (Fig 6A). In contrast, the MMWA treatment could modify the cAMP signaling pathway to exert beneficial effects on insomnia (Fig 6B). Moreover, as shown in S5 Fig, the cAMP signaling pathway was upregulated in the model group compared with the control group. However, it was reversed after the treatment with the MMWA. To verify the effects of MMWA on cAMP levels in PCPA-induced insomnia rats, the concentrations of cAMP were evaluated using ELISA. Compared with the control group, the levels of cAMP were significantly increased in PCPA-induced insomnia rats (P < 0.05). However, the level of cAMP was significantly decreased in the treatment group (P < 0.05) (S6 Fig).

It is noteworthy that, after receiving MMWA treatment, there were significant differences in the metabolite profiles and enrichment KEGG pathways between the control group and the treatment group (P < 0.05) (S6 Fig), indicating that the serum levels of metabolites did not return to a normal state after treatment with the MMWA. This is consistent with our observations of intestinal flora.

### Association of serum metabolites and gut microbiota

As shown in Table 1, the metabolites, such as Amide c18, Benzoyl chloride, Cytosine, and N, n-dimethylarginine were obviously increased in insomnia rats and showed a positive correlation with *Clostridium XlVa* and *Blautia*. Meanwhile, decreased 3-({[(2s)-2,3-dihydroxypropoxy] (hydroxy) phosphoryl}oxy)-2-hydroxypropyl palmitate in the treatment group showed a positive correlation with *Clostridium XlVa* (Table 2).

**Table 1:** Correlation of the metabolite levels with the abundance of gut microbiota in the control and model groups.

Description	Genus	Correlation
Amide c18	*Bifidobacterium*	-0.766758
Amide c18	*Clostridium_XlVa*	0.668651
N, n-dimethylarginine	*Blautia*	0.765262
N, n-dimethylarginine	*Clostridium_XlVa*	0.695278
Benzoyl chloride	*Clostridium_XlVa*	0.721906
Cytosine	*Blautia*	0.697319
2-amino-1,3,4-octadecanetriol	*GpI*	-0.694634
Succinic anhydride	*GpI*	-0.693140
N-acetylserotonin	*Campylobacter*	-0.668781

**Table 2:** Correlation of the metabolite levels with the abundance of gut microbiota in the model and treatment groups.

Description	Genus	Correlation
3-({[(2s)-2,3-dihydroxypropoxy](hydroxy)phosphoryl}oxy)-2-hydroxypropyl palmitate	*Clostridium_XlVa*	0.766920
3-({[(2s)-2,3-dihydroxypropoxy](hydroxy)phosphoryl}oxy)-2-hydroxypropyl palmitate	*Veillonella*	0.578145
4-vinylcyclohexene	*Acinetobacter*	0.758516
Amiloxate	*Elizabethkingia*	0.573944

### MMWA treatment elevates the butyrate level in PCPA-Induced insomnia rats

**Fig 7:**
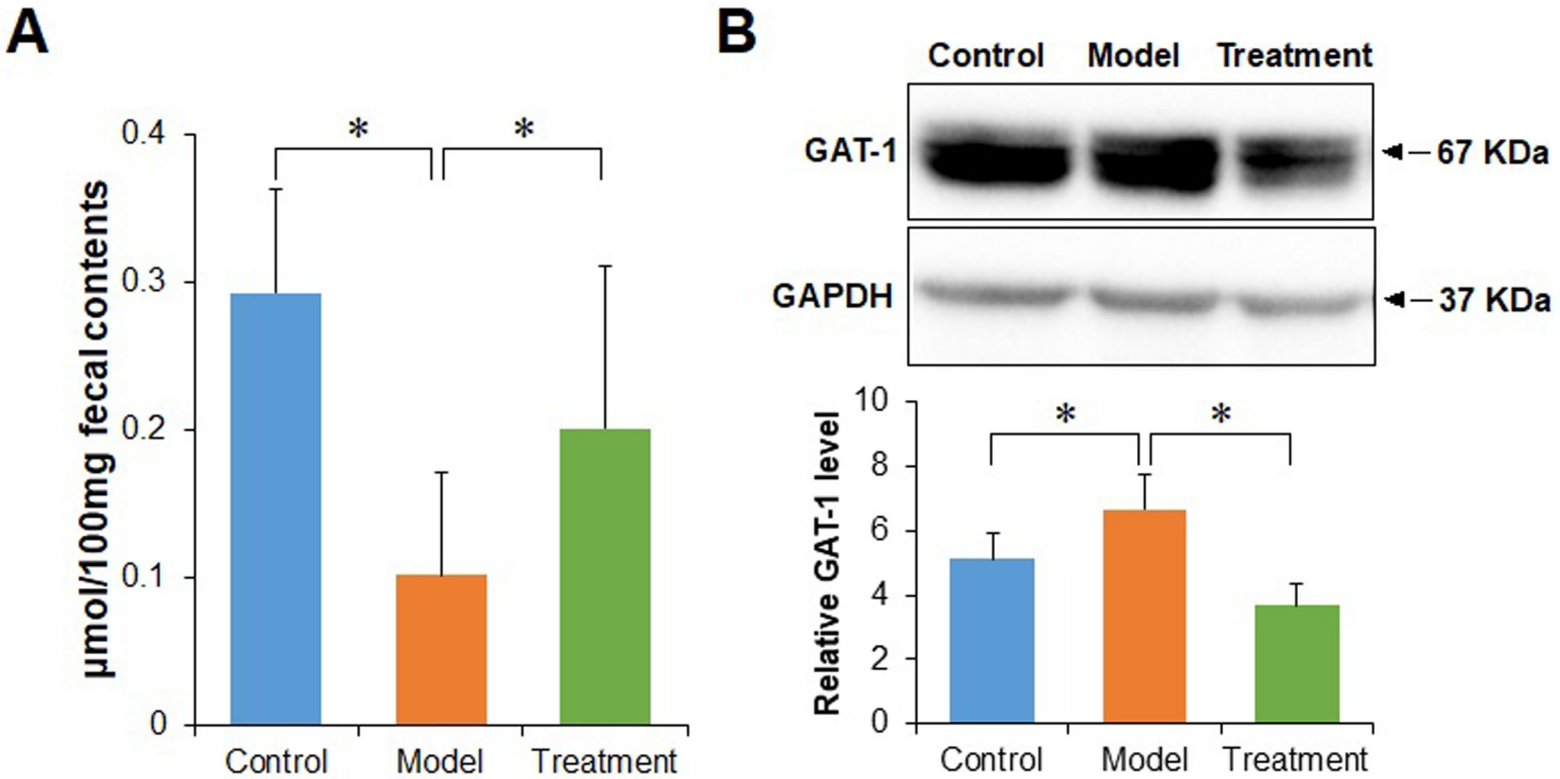
Influence of the MMWA treatment on the level of butyrate and the expression of GAT-1. (A) The level of butyrate in feces. (B) The expression levels of GAT-1 in brain tissues. Values are expressed as the means ± SD, n = 8 rats in each group (*P < 0.05).

The level of fecal butyrate was shown in Fig 7A. The contents of fecal butyrate in the model group tended to decrease compared with the control group (P < 0.05). However, there was higher fecal butyrate in the treatment group than in the model group (P < 0.05). To verify the effects of butyrate on cAMP levels in PCPA-induced insomnia rats, sodium butyrate was administered to the insomnia rats. As shown in S7 Fig, the production of cAMP was increased after PCPA treatment, which was dramatically attenuated by sodium butyrate, suggesting sodium butyrate suppressed PCPA -induced production of cAMP.

### MMWA treatment reduces the GABA transporter 1 (GAT-1) expression in PCPA-induced insomnia rats

As shown in Fig 7B, the rats in the model group showed higher GAT-1 levels compared with the controls (P < 0.05). In contrast, the treatment group showed a lower GAT-1 level than the model group (P < 0.05).

## Discussion

Although the MMWA therapy has been historically used for the treatment of insomnia, the understanding of the underlying mechanisms is still limited [11]. More recently, the results of multiple studies have demonstrated that the gut microbiota and its related metabolites played important roles in the pathogenesis of insomnia [24]. The aim of this study is to evaluate the therapeutic effect of the MMWA and to elucidate its anti-insomnia mechanism from the perspective of intestinal flora and metabolomics.

In our study, the insomnia rat models induced by PCPA were established. We demonstrated that the intervention of the MMWA prevented the loss of body weight and produced a beneficial impact on behavior. The present experiment also showed that the MMWA treatment significantly decreased ACh and NE levels and enhanced 5-HT and GABA levels. It is well known that dysfunction of neurotransmitters is nearly concerned with insomnia. 5-HT and GABA have been found to decrease significantly in brains of insomnia, whereas the levels of ACh and NE increased [25]. Therefore, our observations suggested that the intervention of the MMWA had a satisfactory effect on insomnia.

The present experiment further analyzed gut microbiota composition and diversity based on 16S rRNA gene amplicon sequencing. *Firmicutes* and *Proteobacteria* were the most dominant bacterial phylum, which is consistent with the previous reports [26]. At the genus level, a drastic decrease in the relative abundance of the genera *Lactobacillus* and *Clostridium sensu stricto* were detected in insomnia rats when compared with the controls. *Lactobacillus* species have been widely used as probiotics, which exhibit sleep-improving effects [27]. Additionally, the abundance of *Lactobacillus* and *Clostridium sensu stricto* were associated with the concentration of butyrate [28]. Interestingly, studies have shown that these bacterial species are associated with circadian rhythms [24], and more and more evidence has indicated that butyrate regulates the expression of host circadian clock genes [29]. According to the literature, bacteria tend to affect the host through butyrate [30]. Taken together, these findings suggested that *Lactobacillus* and *Clostridium sensu stricto* might modulate host circadian rhythm at least in part via their metabolite butyrate. Moreover, the abundance of *Romboutsia* was observed to increase in insomnia rats. Members of the genus *Romboutsia* may be involved in dysbiosis of gut microbiota [30], which is consistent with our results. However, the association between *Romboutsia* and insomnia in rats has yet to be elucidated. Interestingly, the intervention of the MMWA caused a reduction in the abundance of *Romboutsia* as well as a significant increase in the abundance of *Lactobacillus* and *Clostridium sensu stricto*, suggesting its potential role in the balance of gut microbiota. Consistently, a previous study showed that acupuncture treatment facilitated *Lactobacillus* on genus level [31].

LEfSe algorithm was used to identify important microbial taxa whose relative abundances differ significantly between groups. At the phylum level, *Proteobacteria* played an essential role in the model group. An increase in the relative abundance of pro-inflammatory *Proteobacteria* in the intestinal microbiota has been observed in rat models of sleep fragmentation [32]. At the family level, *Clostridium XlVa* and *Blautia* characterized the model group. At the order level, *Lachnospiraceae* characterized the model group. *Blautia* and *Lachnospiraceae* have been shown to be negatively correlated with sleep efficiency in humans [33]. To the best of our knowledge, the roles of *Clostridium XlVa* in insomnia have not been reported yet. However, it is interesting to note that *Clostridium XlVa* was enriched in children with autism spectrum disorder [34], suggesting its potential role in neurological pathophysiology. To further verify whether the MMWA treatment rebalances the intestinal flora in the insomnia rat models induced by PCPA, the LDA scores based on LEfSe analysis were obtained between the control and the treatment groups. Surprisingly, the rats in the treatment group were mainly characterized by a higher abundance of *Blautia*. The concept of gut microbiota resilience has been described in the literature [35]. It has been reported that a resilient microbiota will return to its original state of equilibrium after being subjected to a perturbation, whereas a non-resilient microbiota will shift to an alternative stable state [36]. Therefore, we supposed that *Blautia* was quite abundant in the model group, depending on certain host factors, and shifted to an altered state after the MMWA intervention. However, the mechanism of microbiota resilience remains to be elucidated [37].

The alteration of the gut microbiota in insomnia rats was accompanied by the disorders of serum metabolome, as described in other studies [38–40]. Correlation analysis demonstrated that the metabolites elevating in insomnia rats, such as Amide c18, Benzoyl chloride, Cytosine, and N, n-dimethylarginine were positively associated with the abundance of *Clostridium XlVa* and *Blautia*, which characterized the insomnia rats. After treatment with the MMWA, 3-({[(2s)-2,3-dihydroxypropoxy] (hydroxy) phosphoryl}oxy)-2-hydroxypropyl palmitate decreased and showed a positive correlation with *Clostridium XlVa*. These results further confirmed that these bacteria might play an essential role in insomnia.

Metabolomics analysis of serum samples revealed that the MMWA treatment reduced the cAMP signaling pathway in PCPA-induced rat models. A previous study has reported that cAMP is involved in regulating the sleep-wake cycle [41]. The cAMP levels were lowest during rapid eye movement (REM) sleep compared with wakefulness [42], which is consistent with our results. A previous study also found that butyrate could inhibit the cAMP signaling pathway via Gi proteins [43]. We then examined the effect of the MMWA intervention on the butyrate level in insomnia rats. Our results showed that the MMWA significantly increased the content of butyrate. We further confirmed that butyrate treatment attenuated the PCPA-induced increase of the cAMP levels. These results implied that the MMWA inhibited the cAMP signaling pathway at least in part by upregulation of butyrate.

The latest research also indicated that the cAMP signaling pathway played an important role in the regulation of GAT-1 function [44]. It is well known that GABAergic synapse contributed to the development of insomnia. GAT-1 play a key role in regulating GABA levels and is responsible for GABA A receptor (GABAAR)-mediated inhibition [45]. In the present study, we confirmed that the MMWA intervention decreased the expression of GAT-1, which is in agreement with a previous report [44].

## Conclusion

**Fig 8:**
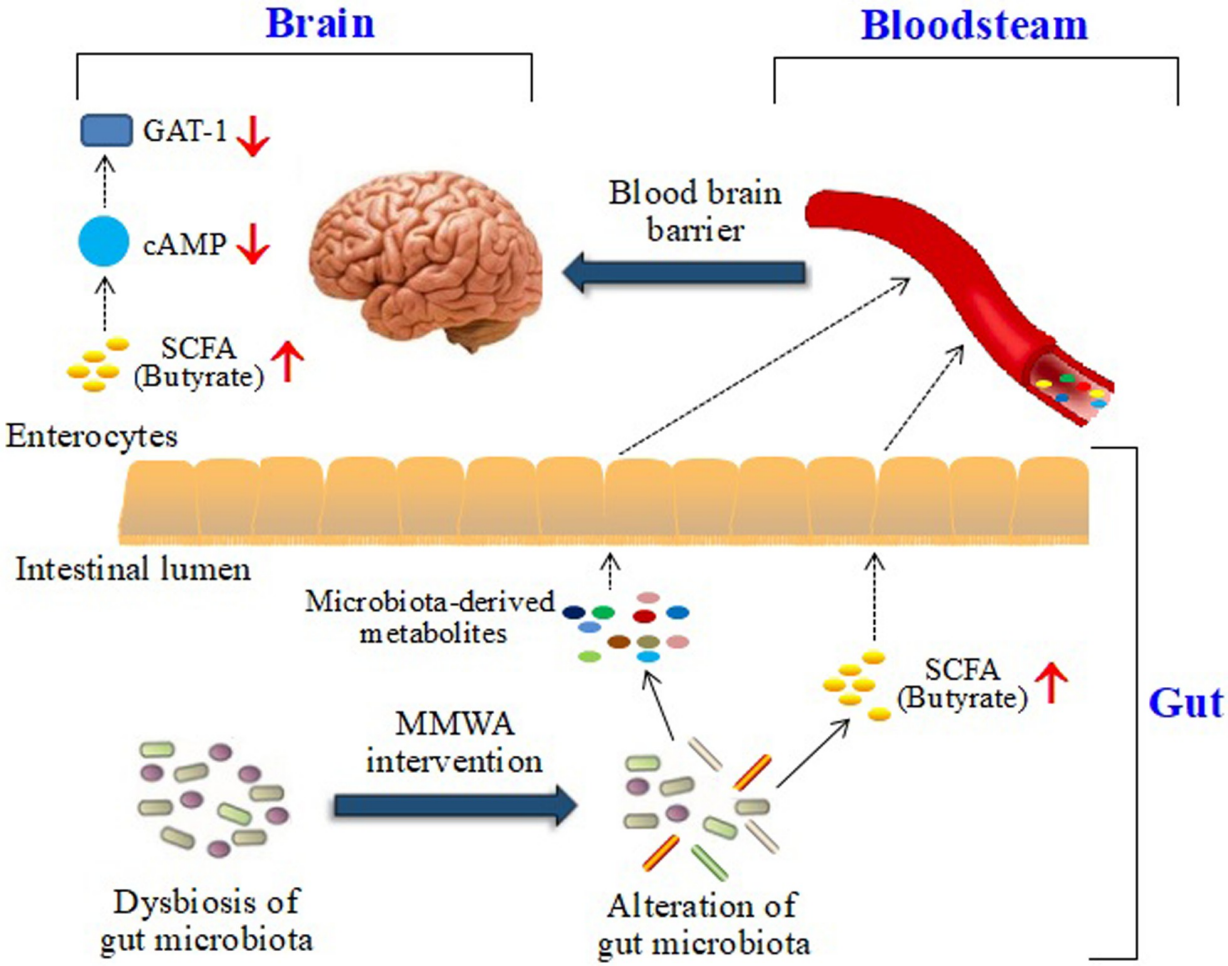
Schematic illustration of potential signaling pathways regulated by the MMWA.

The present study suggested that the MMWA intervention could regulate a variety of microbial genera and metabolites related to insomnia, resulting in the reversal of butyrate-mediated abnormalities of the cAMP signaling pathway and GAT-1 expression (Fig 8). These findings would provide a better understanding of the molecular mechanism underlying the intervention of MMWA as an effective therapy for insomnia.

## Supporting information

S1 Table The number of reads of 16S rRNA gene retained from all samples. (XLSX)

S2 Table The number of OTUs identified from all samples. (XLSX)

S3 Table Number of up- or down-regulated metabolites in the control and model groups. (XLSX)

S4 Table Number of up- or down-regulated metabolites in the model and treatment groups. (XLSX)

S1 Fig Detailed acupoint locations. (TIF)

S2 Fig Species accumulation curve (A) and OTU rank-abundance distribution curve (B) of the intestinal microbiota in rats from the various groups.
(TIF)

S3 Fig Pairwise taxonomic LEfSe analysis of the control and the treatment groups. (TIF)

S4 Fig Analysis of metabolites in the negative ion mode. (TIF)

S5 Fig Number of up- or down-regulated pathways in the control and model groups (A) or in the model and treatment groups (B).
(TIF)

S6 Fig Influence of the MMWA treatment on the levels of cAMP and serum metabolites. (A) Comparison of cAMP levels among three groups. Values are expressed as the means ± SD, n = 8 rats in each group (*P < 0.05). (B) Volcano plot of differential metabolites between the control group and the treatment group. (C) OPLS-DA score plots of serum samples from the control group and the treatment group in positive ion mode. (D) Heatmap analysis of differential metabolites between the control group and the treatment group. (E) KEGG enrichment analysis of differential metabolites between the control group and the treatment group.
(TIF)

S7 Fig Influence of the butyrate on the levels of cAMP in PCPA-induced insomnia rats. (TIF)

S1 Raw image (TIF)

## Abbreviations

Ach: acetylcholine
ELISA: Enzyme-linked immunosorbent assay
FLASH: Fast Length Adjustment of SHort reads
GABA: Gamma-aminobutyric acid
GABAAR: GABA A receptor
GAT-1: Gamma-aminobutyric acid transporter-1
KEGG: Kyoto Encyclopedia of Genes and Genomes
LDA: Linear discriminant analysis
LEfSe: LDA effect size
MMWA: Mongolian medical warm acupuncture
NE: norepinephrine
OTUs: Operational units
PCPA: p-chlorophenylalanine
PICRSt: Phylogenetic Investigation of Communities by Reconstruction of Unobserved States
QC: Quality control
RDP: Ribosomal Database Project
REM: Rapid eye movement
SCFA: Short-chain fatty acid
SD: Sprague-Dawley
5-HT: Serotonin
