# Supplementary figures and images for: Influence of warm acupuncture on gut microbiota and metabolites in rats with insomnia induced by PCPA
Source: PLoS One. 2022 Apr 28;17(4):e0267843. doi: 10.1371/journal.pone.0267843 (PMC9049555; doi:10.1371/journal.pone.0267843)

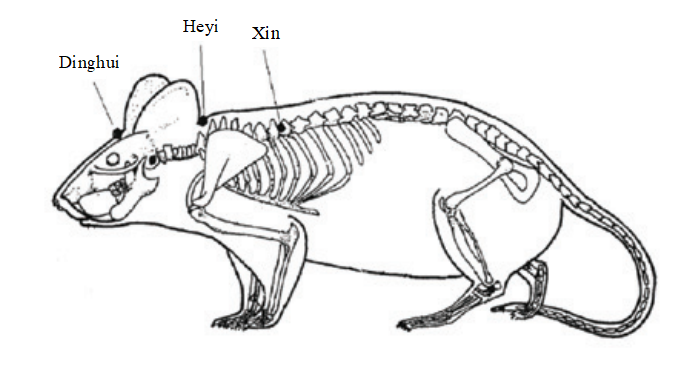

Supplement: S1 Fig — (TIF) [file pone.0267843.s005.tif]

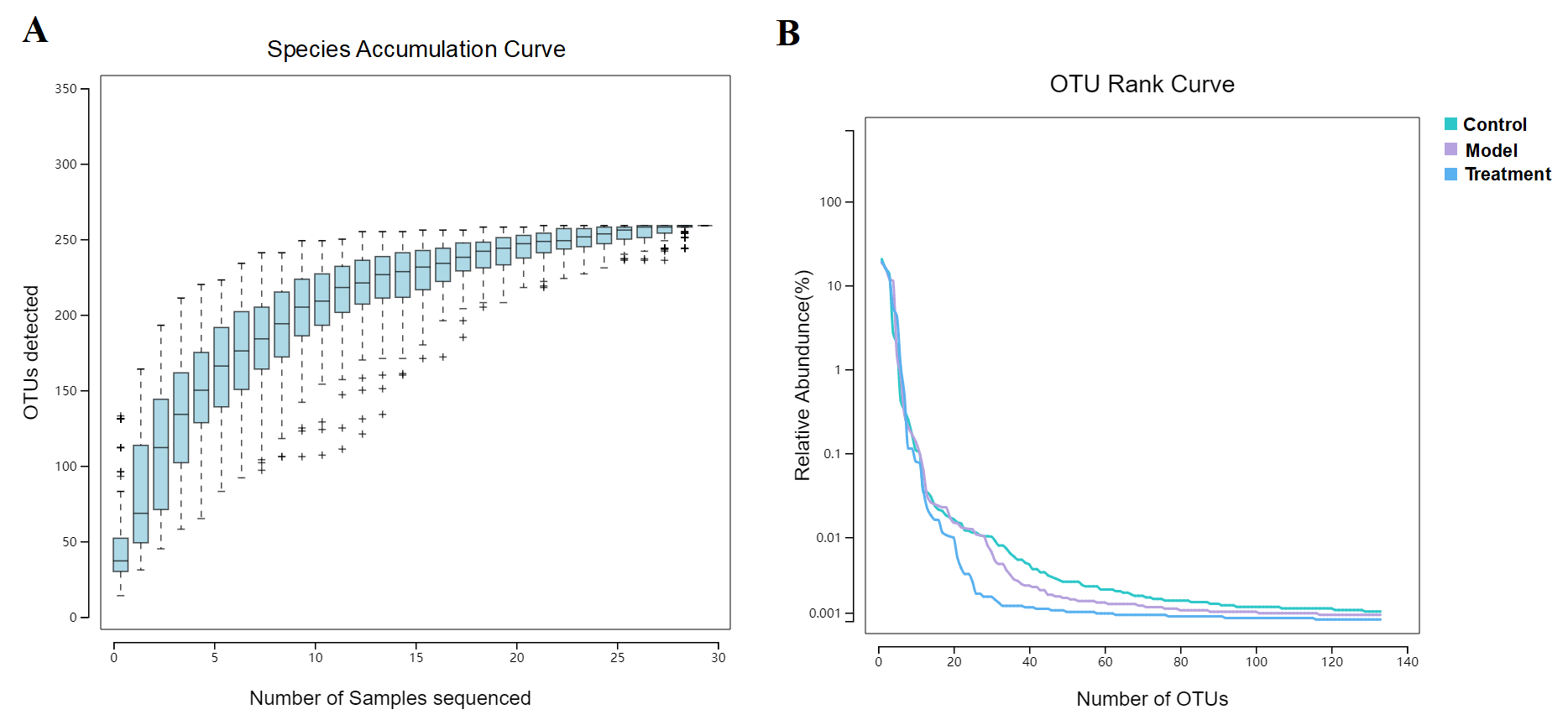

Supplement: S2 Fig — Species accumulation curve (A) and OTU rank-abundance distribution curve (B) of the intestinal microbiota in rats from the various groups. (TIF) [file pone.0267843.s006.tif]

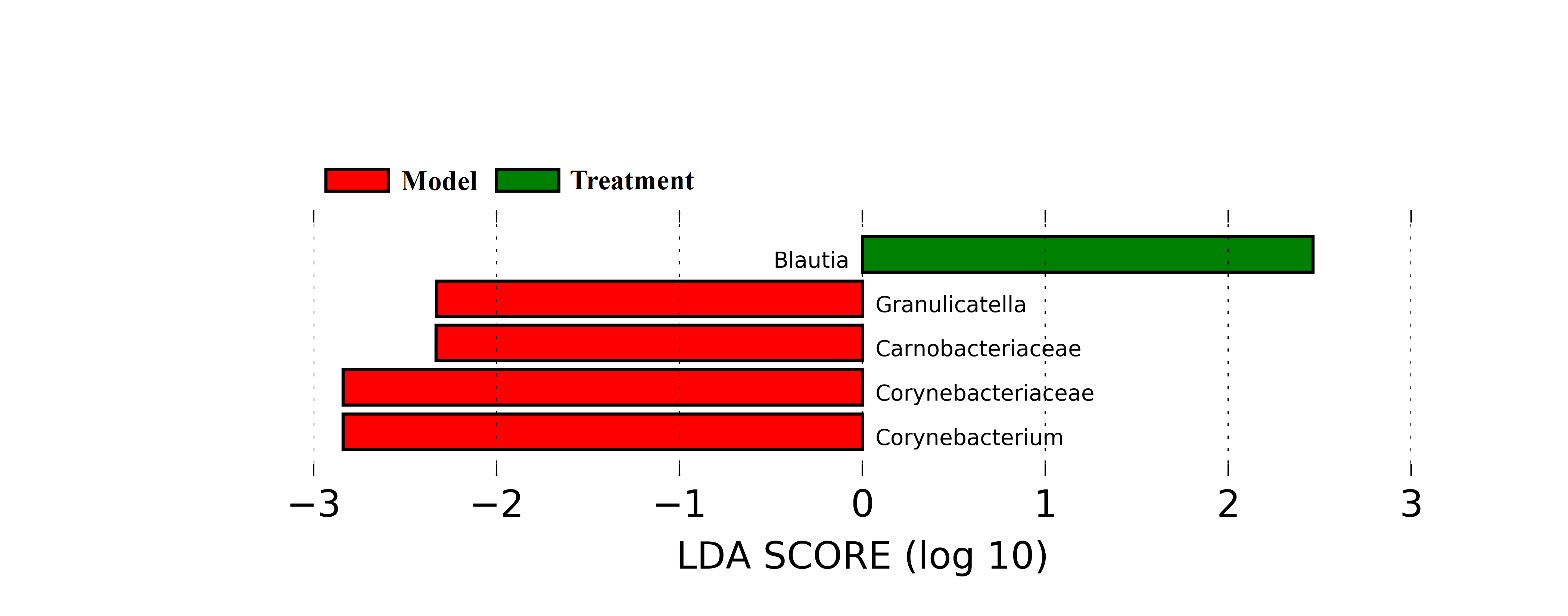

Supplement: S3 Fig — (TIF) [file pone.0267843.s007.tif]

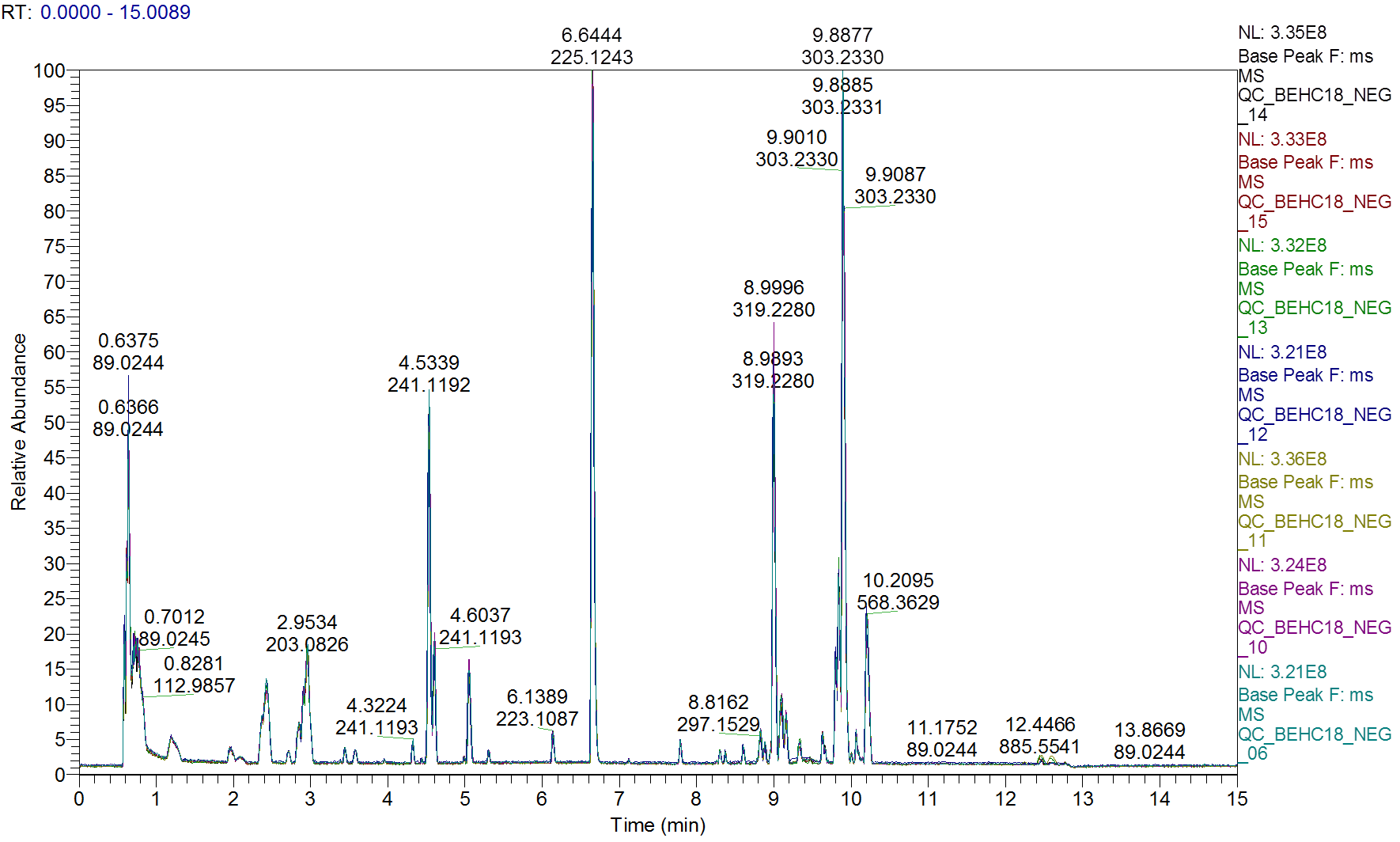

Supplement: S4 Fig — (TIF) [file pone.0267843.s008.tif]

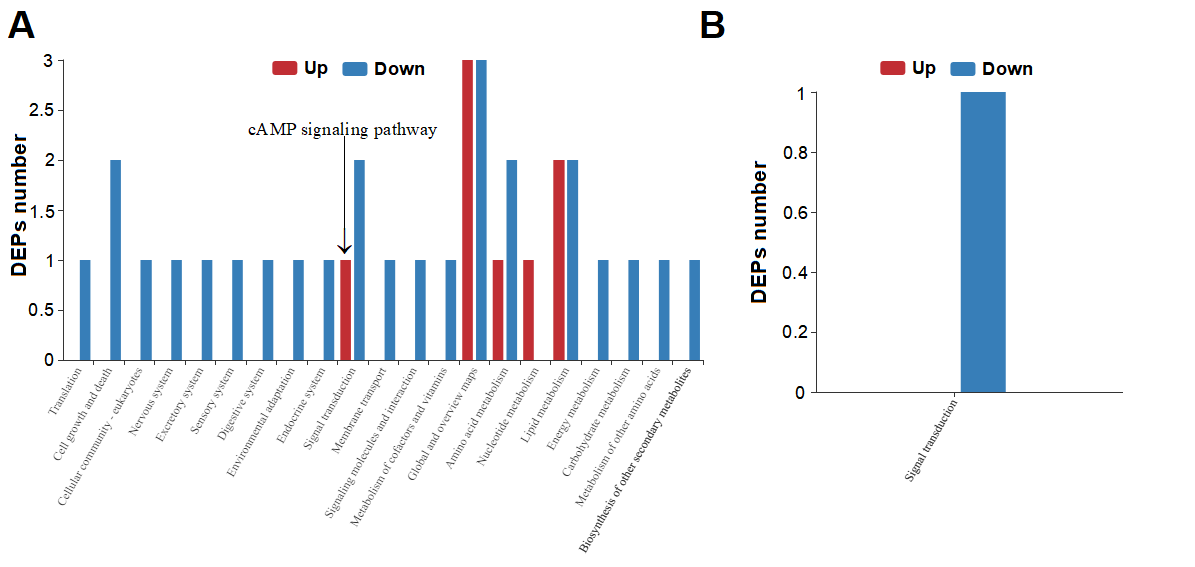

Supplement: S5 Fig — Number of up- or down-regulated pathways in the control and model groups (A) or in the model and treatment groups (B). (TIF) [file pone.0267843.s009.tif]

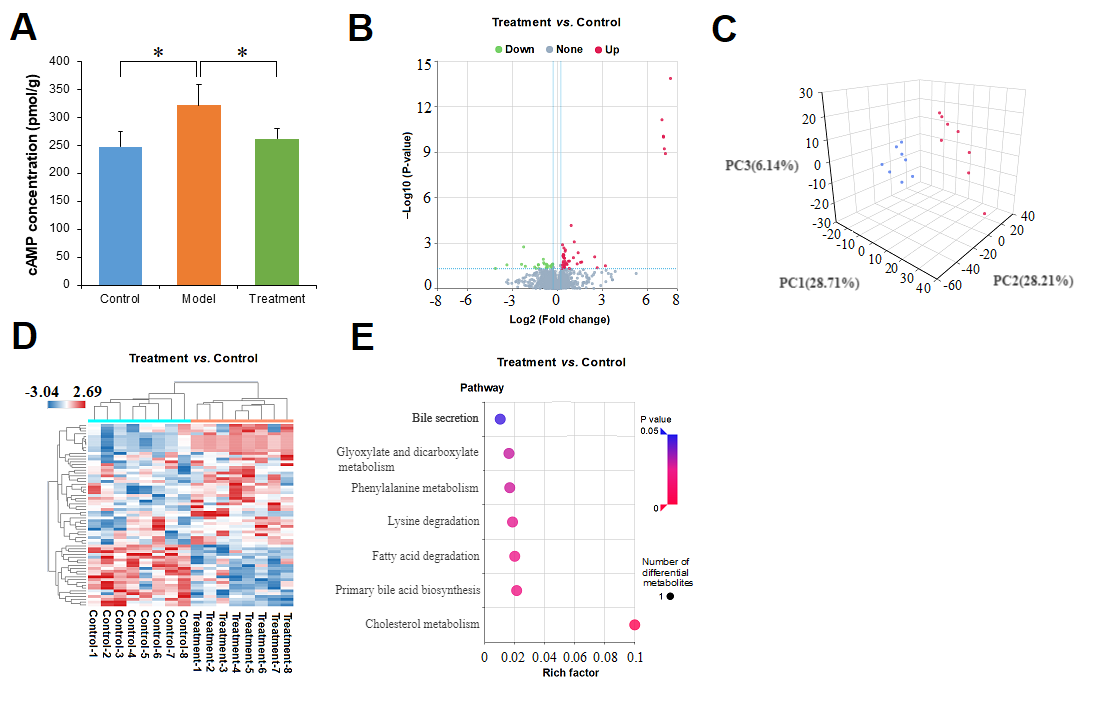

Supplement: S6 Fig — (A) Comparison of cAMP levels among three groups. Values are expressed as the means ± SD, n = 8 rats in each group (*P < 0.05). (B) Volcano plot of differential metabolites between the control group and the treatment group. (C) OPLS-DA score plots of serum samples from the control group and the treatment group in positive ion mode. (D) Heatmap analysis of differential metabolites between the control group and the treatment group. (E) KEGG enrichment analysis of differential metabolites between the control group and the treatment group. (TIF) [file pone.0267843.s010.tif]

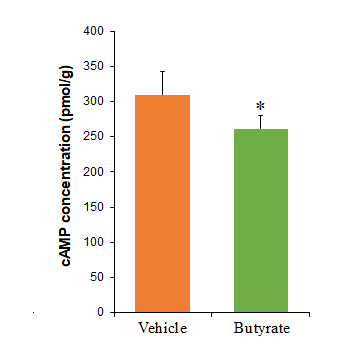

Supplement: S7 Fig — (TIF) [file pone.0267843.s011.tif]

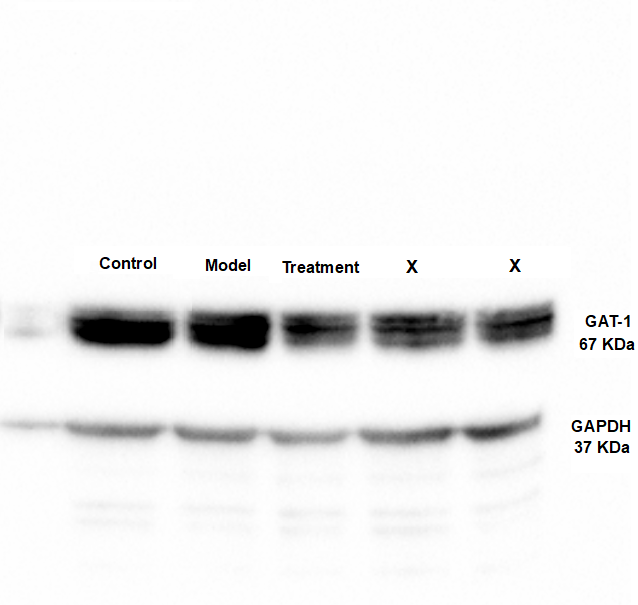

Supplement: S1 Raw image — (TIF) [file pone.0267843.s012.tif]
